# Health literacy: exploring disparities among college students

**DOI:** 10.1186/s12889-019-7781-2

**Published:** 2019-10-29

**Authors:** Jehad A. Rababah, Mohammed M. Al-Hammouri, Barbara L. Drew, Mohammed Aldalaykeh

**Affiliations:** 1 Adult Health Nursing Department, Faculty of Nursing, Jordan University of Science and Technology, P.O. Box 3030, Irbid, 22110 Jordan; 2 Community and Mental Health Department, Faculty of Nursing, Jordan University of Science and Technology, Irbid, Jordan; 3 College of Nursing, Kent State University, Kent, OH USA

**Keywords:** Health literacy, College students, Health literacy questionnaire, HLQ, Health disparities

## Abstract

**Background:**

Health literacy is a multidimensional concept that is considered a primary public health concern. This concept is often neglected in research, and the evidence regarding health literacy in college students is limited. The aim of this study was two-fold: to determine the needs and limitations of health literacy among college students and to explore the relationships among the nine dimensions of health literacy and sociodemographic factors, including age, gender, nationality, year of study, field of study, smoking status, history of chronic diseases, use of on-campus gym, and the intention to exercise on-campus.

**Methods:**

This study was conducted using a cross-sectional approach. A total of 520 college students participated in this study. The average age was 21.03 years (SD = 2.29), with 47.5% males and 52.5% females. Data were collected using a demographics questionnaire and the Health Literacy Questionnaire (HLQ). In addition to descriptive statistics, multivariate analysis of variance (MANOVA) and follow-up analyses were performed to explore any statistically significant mean differences among levels of health literacy and sociodemographic factors.

**Results:**

The levels of health literacy on the nine HLQ scales were lower than the levels reported in the literature. Multivariate analysis showed a significant effect of age, gender, smoking status, year of study, and field of study on the level of health literacy. Follow-up analyses revealed that female students, students from the health-related faculties, and those who do not smoke have higher levels of health literacy compared to their counterparts. A detailed comparison between the levels of the HLQ scales was made based on students’ demographic characteristics. The field of study had the most prominent effect on the level of college students’ health literacy; mean differences were statistically significant (*p* < .001), and effect sizes were large (ranging from .66 to 1.35 for the nine scales of the HLQ).

**Conclusion:**

College students’ health literacy is influenced by demographic characteristics. Such variations could amplify some of the existing health disparities. The implications of the findings on health, health promotion, and interprofessional education are discussed.

## Background

### Health literacy defined

Health literacy is an important public health concern that is often neglected in research [1]. There is no definite agreement on the definition of health literacy. A widely used definition of health literacy is “the degree to which individuals can obtain, process, and understand the basic health information and services they need to make appropriate health decisions” (2 p1). The World Health Organization (WHO) described health literacy as “both a means and an outcome of actions aimed at promoting the empowerment and participation of people in their communities and of people in their health care” (1 p6). Sørensen and colleagues [2] provided a list of the most commonly used definitions of health literacy. Generally speaking, the definitions address the importance of having both sound cognitive abilities and competent skills to obtain, understand, and utilize health information to make appropriate health-related decisions. The ultimate outcome of being health literate is to manage and promote health.

### Limited health literacy

The level of health literacy among individuals and groups ranges from limited to adequate. The U.S. Department of Health and Human Services asserted that limited health literacy is a common issue [3]. Factors that predispose an individual to have a limited health literacy include a lower level of education, older age, and migration [1]. Moreover, people with poor socioeconomic status and those who are considered minorities are at greater risk of having limited health literacy [3]. Statistics regarding the epidemiology of limited health literacy, available mainly from the U.S. and the European Union (EU), provide worrying numbers. Based on the National Assessment of Adult Literacy (NAAL), 36% of the U.S. population had basic or below basic health literacy [3]. The situation in the EU is more disturbing, with almost half of Europeans found to have limited health literacy [1]. This was reported in a survey conducted in eight countries: Austria, Bulgaria, Germany, Greece, Ireland, the Netherlands, Poland and Spain [4]. The populations studied in these countries represent diverse cultures and nationalities.

To the best of the authors’ knowledge, such data from developing countries are limited. However, the possibility of having limited health literacy in developing countries could be higher considering the relatively lower socioeconomic status and education levels. Limited health literacy adversely affects self-management of health, especially for those with or at risk for chronic diseases [1]. In addition, it results in poorer health outcomes and a higher likelihood of hospitalizations and hinders preventive care [3, 5]. The individual’s ability to adopt a healthy lifestyle diminishes and the risk of having unhealthy behaviors increases when health literacy is limited [1]. The end result of having limited health literacy is a substantial rise in healthcare costs [1].

In addition, there are some common procedural limitations of health literacy studies. A review of the related literature showed that the focus of health literacy research has been on measuring functional health literacy, that is, the ability to read and understand health information [1, 6]. Making an informed decision extends beyond the level of merely comprehending such information. Another related issue is that the administration of shortened versions of health literacy measurement tools increases the risk of ignoring key aspects of multidimensional health literacy. The following section will discuss common health problems experienced by college students and summarize the literature regarding health literacy among them. To overcome such procedural issues, we used the Health Literacy Questionnaire (HLQ) [7] in this study. The HLQ was developed following a validity-driven approach and covers various dimensions of measuring health literacy. According to the WHO, the HLQ is recommended for use in the identification of health literacy strengths and limitations [8]. Thus, the HLQ was consistent with one of the objectives of the current study.

### College students’ health

Throughout their course of study, college students experience a unique transition from high school to adulthood. Meleis defined transition as “a passage from one fairly stable state to another fairly stable state, and it is a process triggered by change” (9 p11). Going through such developmental transition brings about milestone changes in college students’ lives. According to the literature, college students worldwide experience high levels of psychological distress [9–11] that exceed the levels experienced by nonstudents [12]. There are many factors, such as individual differences and environmental influences, that make someone’s experience with the transition process different [13]. However, there are common circumstances experienced by the majority of college students that could negatively affect students’ health. Examples include academic responsibilities, building new social networks, financial concerns, and living adjustments [10, 11]. In addition, a report from the Centers for Disease Prevention and Control (CDC) indicates that consuming unhealthy food, stress, sleep deprivation, and peer pressure to use harmful substances are all examples of the circumstances that affect college students’ health [14].

Colleges are considered ideal settings for starting health promotion programs [15, 16]. The uniqueness of colleges lies mainly in the availability of human and physical resources to foster adopting healthy lifestyle changes. Surprisingly, the results of the American College Health Association-National College Health Assessment (ACHA-NCHA) showed that significant proportions of undergraduate students have not received information regarding specific health topics from their higher education institutions [17]. At the same time, the students reported their need to receive information regarding health. Table 1 summarizes the most alarming findings from the ACHA-NCHA. For example, 76.9% of students reported that they had not received information about the issue of “Sleep Difficulty”. Meanwhile, the majority of students (67.5%) showed interest in learning about this issue. The significance of these findings is two-fold; they 1) address the responsibility of higher education institutions and 2) highlight the topics in which college students are interested in obtaining more information. Clearly, these topics are of great importance for different dimensions of health, including physical, psychological, psychiatric, social, and reproductive health.

**Table 1:** Summary of the ACHA-NCHA Results^a^

Health Topic	Percentage of students who have not received information	Percentage of students interested in learning about the topic
Problem use of internet/computer games	81.7	31.3
Sleep difficulties	76.9	67.5
Eating disorders	68.1	46.6
Grief and loss	65.4	54.7
Injury prevention	65.0	51.8
Tobacco use	58.5	34.8
Relationship difficulties	55.3	53.7
Pregnancy prevention	51.8	46.3
Suicide prevention	46.0	60.5
Physical activity	41.0	63.9

*ACHA-NCHA* American College Health Association-National College Health Assessment

^a^These results were extracted from the ACHA-NCHA (2018) report

### Health literacy among college students

The percentages presented in the table raise a key question: how much do we know about health literacy among college students? Many studies have shown that college students have adequate levels of health literacy [18–20]. Other studies conducted mainly outside the U.S., on the other hand, reported inadequate levels of health literacy [21, 22]. Analysis of the relevant literature showed that many of the studies conducted among college students share the common drawbacks, mentioned earlier, of studying health literacy among general populations. Additionally, a central recommendation of many studies was the importance of investigating potential relationships between sociodemographic variables and the level of health literacy [18, 20].

*Exploring Health Literacy in Tertiary Students: An International Study* is a global project that was launched in 2014 to measure the level of health literacy among college students studying the health professions [23]. This project, with data collected from 19 universities in 10 countries worldwide using the HLQ, has led to significant advances in studying health literacy in college students. Data from different participating countries (namely, the U.S., Denmark, and China) have already been published. In the U.S., Vamos and colleagues intended to explore the effect of sociodemographic variables on health literacy [24]. They concluded that college students’ age, gender, and parents’ education affect health literacy. The Danish study also examined the effect of sociodemographic variables on the level of health literacy among college students from four different health professions [25]. The authors reported a positive correlation between parents’ education and health literacy and found that public health students had higher scores on the HLQ scales. Regarding the data from China, college students’ health literacy varied based on students’ gender, year and field of study, socioeconomic status, area of residence (urban vs. rural), and parents’ education [26]. Comparable findings were also reported in a study that was conducted in Turkey using the Adult Health Literacy Scale [27].

The conclusions drawn from these studies were similar and emphasized the importance of exploring the impact of college students’ sociodemographic characteristics on health literacy around the world. Therefore, this study was conducted to 1) determine the needs and limitations of health literacy among four-year college students and 2) explore the relationships among the nine dimensions of health literacy and sociodemographic factors, including age, gender, nationality, year of study, field of study, smoking status, history of chronic diseases, use of on-campus gym, and the intention to exercise on-campus. These sociodemographic factors were selected based on the evidence regarding their relationship with health literacy in other higher education settings and populations such as those with chronic illnesses [24–30].

## Methods

### Design and setting

This study was conducted using a cross-sectional, exploratory design. It was conducted at a large public university in northern Jordan. The current study is a part of a larger ongoing project conducted to explore health promotion and self-care among university students.

### Sampling and participants

After obtaining the Institutional Review Board (IRB) approval from Jordan University of Science and Technology, four-year college students were invited to participate in this study. A purposive sampling approach was used to recruit a sample, which was as representative as possible, of the accessible population. Two criteria were considered during the recruitment of participants to enhance the representativeness of the sample: 1) recruiting students from the health-related and other fields of study proportional to the actual numbers of students in these fields and 2) recruiting students who represent the different years of study. A written informed consent was obtained from each participant after explaining the study, its purpose, and the potential benefits. A total of 520 participants, with a response rate of 79.3%, completed a demographics questionnaire and the health literacy questionnaire (HLQ) [29]. The average age of participants was 21.03 years (SD = 2.29), with 47.5% males and 52.5% females. A summary of the participants’ characteristics is presented in Table 2.

**Table 2:** Participants Demographic Characteristics

Variable	Total (*N* = 520)	
	N	Percentage
Gender		
Male	247	47.5
Female	273	52.5
Field of study		
Health-related	308	59.2
Others (engineering, sciences)	212	40.8
Year of study		
First	101	19.4
Second	149	28.7
Third	107	20.6
Fourth	133	25.6
Fifth-sixth	30	5.8
Nationality		
Jordanian	495	95.2
International	25	4.8
Smoking		
No	385	74
Yes	135	26
Diagnosed with chronic diseases		
No	496	95.4
Yes	24	4.6
Use the on-campus gym		
No	398	76.5
Yes	122	23.5
Intention to exercise on-campus		
No	183	35.2
Yes	337	64.2

### Data collection tools

A demographics questionnaire was developed for this study and included items regarding the age, gender, field of study, year of study, smoking status, existence of chronic diseases, allergies, vaccination history, and physical activity of participants (See Additional file 1). We also used the Arabic version of the HLQ, which was provided by the developers of the tool. Up to this point there are no studies that investigated the psychometrics of the Arabic version of the HLQ yet. However, according to the developers of the HLQ, this tool has been translated using a rigorous process to ensure consistency of the translated versions with the original HLQ in terms of the psychometric properties.

#### Health literacy questionnaire

The HLQ [7] is a 44-item tool used to measure health literacy. It was developed following a validity-driven approach utilizing data from the general population, patients, healthcare providers, and policy makers. Items of the HLQ cover nine areas pertinent to the challenges and needs of people. These areas are reflected in nine scales of the HLQ: 1) Feeling understood and supported by healthcare providers (four items); 2) Having sufficient information to manage my health (four items); 3) Actively managing my health (five items); 4) Social support for health (five items); 5) Appraisal of health information (five items); 6) Ability to actively engage with healthcare providers (five items); 7) Navigating the healthcare system (6 items); 8) Ability to find good health information (five items); and 9) Understand health information (five items). The first five scales contain items with responses ranging from one (strongly disagree) to four (strongly agree). The remaining scales, six through nine, include items with five responses: 1 = cannot do or always difficult, 2 = usually difficult, 3 = sometimes difficult, 4 = usually easy, and 5 = always easy. There is no total score for the HLQ; instead, it is recommended to calculate the scores for nine scales to examine the needs and limitations of health literacy [7]. The total score of a given scale is calculated as the mean average of the items within that scale. Possible total scores range from one to four in the first five scales and one to five in scales six through nine. All scales were administered in combination in the current study to collect the full range of information on health literacy among college students.

The factor structure of the HLQ and its scales has been examined using confirmatory factor analysis and exploratory structural equation modeling [7]. Osborne and colleagues found that composite reliability was above .80 for all scales except for the “Appraisal of Health Information” scale. In the current study, Cronbach’s alpha was calculated for each scale. The results showed that seven out of the nine scales had Cronbach’s alpha values above the recommended value of .70, with a range from .70 to .82. The values for two scales were below that value: “Feeling Understood and Supported by Healthcare Providers” (Cronbach’s α = .61) and “Having Sufficient Information to Manage My Health” (Cronbach’s α = .65).

### Data analysis

All data were analyzed using SPSS (version 23). Descriptive statistics and frequencies were performed. As noted earlier, the HLQ provides nine separate scores for each scale, and these scores were considered the dependent variables (DVs). Age, gender, field of study, year of study, nationality, smoking status, being diagnosed with chronic disease(s), use of on-campus gym, and intention to exercise on-campus were all treated as the independent variables (IVs). Based on that, multivariate analysis of variance (MANOVA) was conducted. Running MANOVA in SPSS yields four multivariate outcomes: Pillai’s Trace, Wilks’ Lambda, Hotelling’s Trace, and Roy’s Largest Root. The decision was made to use Roy’s Largest Root for three reasons: 1) some of the IVs had more than two groups, 2) the sample sizes of the groups were not equal, and 3) the distribution of the data was not platykurtic [31, 32]. In the case of having significant multivariate results of the Roy’s Largest Root statistic, follow-up analyses using ANOVA and independent t-tests were performed to detect the source of the statistically significant mean differences in the nine scales of the HLQ. Because of the different sample sizes, Gabriel’s test was used to interpret the results of follow-up ANOVAs [31, 32]. The *P* values were adjusted in these follow-up analyses to avoid inflation of Type I error (see the Results section).

## Results

To obtain an overall idea about participants’ health literacy needs and challenges, descriptive statistics of the nine HLQ scales were performed first. The “Social Support for Health” scale had the highest average score (Mean = 2.87, SD = .56) for scales one through five. For the remaining scales, the “Ability to Find Good Health Information” had the highest average (Mean = 3.57, SD = .74). The average scores, standard deviations, and ranges of the HLQ scales are presented in Table 3. It is worth noting that this table is not intended for comparison between the scores of the nine HLQ scales. Rather, it is just to give the reader an overall idea about the levels of health literacy among the study sample.

**Table 3:** Descriptive Statistics of the HLQ scales (*N* = 520)

Scale	Mean (SD)	Minimum	Maximum
Scales with possible total score of 1–4			
Feeling understood and supported by healthcare providers	2.54 (.58)	1	4
Having sufficient information to manage my health	2.71 (.57)	1	4
Actively managing my health	2.7 (.54)	1	4
Social support for health	2.87 (.56)	1	4
Appraisal of health information	2.79 (.58)	1	4
Scales with possible total score of 1–5			
Ability to actively engage with healthcare providers	3.48 (.78)	1	5
Navigating the healthcare system	3.42 (.81)	1	5
Ability to find good health information	3.57 (.74)	1	5
Understand health information well enough to know what to do	3.53 (.74)	1	5

*HLQ* health literacy questionnaire, *SD* standard deviation

### Multivariate and univariate analyses

Assumptions were first checked before conducting and interpreting MANOVA. Considering the size of our sample, histograms were used to check for normal distribution [32], and they are provided as a supplementary file (See Additional file 2). According to Mayers, a multivariate effect exists when there is a correlation between the DVs with acceptable positive correlations ranging from .30 to .90 [32]. Prior to performing MANOVA, the correlations between the HLQ scales were first examined using Pearson’s r correlation. The results showed that all correlations fell within that range (r = .46 to .82). The homogeneity of variance-covariance matrices was evidenced by having a nonsignificant Box’s Test (*p* = .082). Levene’s Test statistic was also not significant (*p* values for the nine scales ranged from .06 to .88), indicating homogeneity of between-group variance.

MANOVA analysis showed statistically significant multivariate differences in the nine scales of the HLQ based on participants’ age (Roy’s Largest Root = .076, F {15, 492} = 2.49, *p* = .001), gender (Roy’s Largest Root = .037, F {9, 484} = 1.97, *p* = .041), smoking status (Roy’s Largest Root = .04, F {9, 484} = 2.17, *p* = .023), year of study (Roy’s Largest Root = .052, F {9, 488} = 2.81, *p* = .003), and field of study (Roy’s Largest Root = .49, F {9484} = 26.54, *p* < .001). The rest of the IVs did not have a statistically significant multivariate effect on the scales of the HLQ; consequently, they were not considered in the follow-up analyses. Regarding the interaction effects, no statistically significant effects were found. Univariate results revealed that two IVs, that is, smoking status and field of study, had statistically significant effects on the DVs (see Table 4). Partial Eta Squared (Partial η^2^) was used to posteriori calculate the effect sizes presented in the table. Cohen’s guidelines were used to interpret the resulting effect sizes: small <.25, medium = .25 to .40, and large >.40 [33].

**Table 4:** Univariate Analysis^a^

IV	DV	F	Significance	Partial Eta Squared	Effect Size^b^
Smoking	HPS	2.65	.104	.005	.07
	HSI	11.55	.001	.023	.15
	AMH	6.29	.012	.013	.11
	SS	5.55	.019	.011	.11
	CA	16.15	< .001	.032	.18
	AE	3.71	.055	.007	.08
	NHS	5.61	.018	.011	.11
	FHI	3.71	.055	.007	.08
	UHI	4.47	.035	.009	.10
Field of Study	HPS	206.65	< .001	.296	.65
	HSI	101.66	< .001	.171	.45
	AMH	77.97	< .001	.137	.40
	SS	52.06	< .001	.096	.33
	CA	75.18	< .001	.133	.39
	AE	82.01	< .001	.143	.41
	NHS	75.64	< .001	.133	.39
	FHI	47.28	< .001	.088	.31
	UHI	79.73	< .001	.139	.40

*Abbreviations*: *IV* independent variable, *DV* dependent variable, *HPS* feeling understood and supported by healthcare providers, *HSI* having sufficient information to manage my health, *AMH* actively managing my health, *SS* social support for health, *CA* appraisal of health information, *AE* ability to actively engage with healthcare providers, *NHS* navigating the healthcare system, *FHI* ability to find good health information, *UHI* understand health information

^a^Degrees of freedom = (1, 492)

^b^ Calculated posteriori using G*Power (Version 3.1.9.2)

### Post hoc and follow-up analyses to locate the source of main effects

Post hoc analysis, using Gabriel’s statistic, showed the following statistically significant results: 1) second-year students had lower scores on the “Feeling understood and supported by healthcare providers” scale than fifth- and sixth-year students (*p* = .01); 2) third-year students had lower scores on the “Feeling understood and supported by healthcare providers” scale than fifth- and sixth-year students (*p* = .025); 3) third-year students had lower scores on the “Having sufficient information to manage my health” scale than fifth- and sixth-year students (*p* = .032); 4) first-year students had lower scores on the “Appraisal of health information” scale than fifth- and sixth-year students (*p* = .001); 5) second-year students had lower scores on the “Appraisal of health information” scale than fifth- and sixth-year students (*p* = .001); 6) third-year students had lower scores on the “Appraisal of health information” scale than fifth- and sixth-year students (*p* = .001); 7) fourth-year students had higher scores on the “Appraisal of health information” scale than fifth- and sixth-year students (*p* = .004); 8) third-year students had lower scores on the “Ability to find good health information” scale than fourth-year students (*p* = .016); 9) second-year students had lower scores on the “Understand health information” scale than fourth-year students (*p* = .01); and 10) third-year students had lower scores on the “Understand health information” scale than fourth-year students (*p* = .024).

A comparison of the means of the DVs based on the IVs that exhibited significant multivariate effects was made. There was a need to run five separate t-tests. Therefore, *p* values were adjusted in these follow-up analyses as follows: .05 ÷ 5 = .01. The results of these analyses are presented in Table 5. The table also includes the effect sizes pertinent to each IV and the lower and upper bounds of the 95% confidence interval (CI) of difference. The effect sizes were calculated posteriori using G*Power (Version 3.1.9.2).

**Table 5:** Follow up analyses to detect the source of differences

DV IV	HPS	HSI	AMH	SS	CA	AE	NHS	FHI	UHI
Age									
≤ 21.03 (*n* = 234)	2.56	2.70	2.71	2.90	2.80	3.40	3.46	3.60	3.50
> 21.03 (*n* = 286)	2.52	2.72	2.70	2.84	2.80	3.50	3.40	3.60	3.50
Effect size	.05	.03	.01	.09	.02	.14	.07	.01	.01
(95% CI)	(−.07 to .13)	(−.12 to .08)	(−.09 to .10)	(−.04 to .15)	(−.11 to .09)	(−.02 to .25)	(−.08 to .20)	(−.12 to .14)	(−.12 to .14)
Gender									
Male (*n* = 247)	2.50	2.67	2.70	2.80*	2.73	3.44	3.32*	3.47*	3.43*
Female (*n* = 273)	2.57	2.73	2.72	2.92*	2.85	3.52	3.51*	3.66*	3.62*
Effect size	.12	.07	.04	.24	.21	.10	.24	.26	.26
(95% CI)	(−.17 to .03)	(−.14 to .05)	(−.12 to .07)	(−.23 to −.04)	(−.22 to −.02)	(−.21 to .06)	(−.33 to −.06)	(−.31 to −.06)	(−.31 to −.06)
Field of Study									
Health-related (*n* = 308)	2.80**	2.90**	2.88**	3.01**	2.98**	3.74**	3.64**	3.77**	3.77**
Non-health (*n* = 212)	2.16**	2.43**	2.47**	2.65**	2.52**	3.11**	3.04**	3.28**	3.17**
Effect size	1.35	.92	.81	.66	.85	.84	.86	.68	.87
(95% CI)	(.56 to .73)	(.39 to .57)	(.32 to .49)	(.26 to .45)	(.37 to .55)	(.49 to .75)	(.51 to .77)	(.36 to .61)	(.48 to .72)
Year of Study									
1st or 2nd year (*n* = 250)	2.52	2.70	2.71	2.85	2.77	3.47	3.39	3.54	3.47
≥ 3rd year (*n* = 270)	2.56	2.72	2.71	2.89	2.81	3.49	3.45	3.60	3.59
Effect size	.07	.03	.01	.05	.06	.02	.07	.07	.16
(95% CI)	(−.14 to .06)	(−.11 to .08)	(−.09 to .10)	(−.12 to .07)	(−.13 to .07)	(−.15 to .12)	(−.20 to .08)	(−.18 to .07)	(−.25 to .01)
Smoking									
No (*n* = 385)	2.57	2.76**	2.75*	2.91*	2.86**	3.53	3.49*	3.63*	3.59*
Yes (*n* = 135)	2.44	2.56**	2.60*	2.72*	2.60**	3.35	3.22*	3.40*	3.36*
Effect size	.23	.29	.27	.95	.45	.23	.33	.31	.30
(95% CI)	(.02 to .25)	(.09 to .31)	(.04 to .25)	(.08 to .30)	(.15 to .38)	(.03 to .34)	(.12 to .43.)	(.08 to .37)	(.08 to .37)

*Abbreviations*: *IV* independent variable, *DV* dependent variable, *HPS* feeling understood and supported by healthcare providers, *HSI* having sufficient information to manage my health, *AMH* actively managing my health, *SS* social support for health, *CA* appraisal of health information, *AE* ability to actively engage with healthcare providers, *NHS* navigating the healthcare system, *FHI* ability to find good health information, *UHI* understand health information, *CI* confidence interval (of difference)

* *p* value < .01

** *p* value < .001

## Discussion

The current study was conducted in the hope of generating university-specific information regarding health literacy among college students. The authors conducted this research among college students for different reasons. Most importantly, the literature regarding health literacy, measured by the HLQ, in this population is scarce. College students spend a significant amount of time in a stressful environment, and they are at high risk for engaging in unhealthy behaviors. Having an adequate level of health literacy can help college students make appropriate decisions about their health. On the other hand, making inappropriate decisions and engaging in unhealthy behaviors, such as unhealthy eating [34] and getting less than the recommended amount of physical activity [35], negatively impacts academic achievement. The long-term effect of such health problems is detrimental as they increase the risk for life-threatening, chronic problems such as cardiovascular disease [36], metabolic syndrome [37], and mental health problems [38]. Therefore, understanding the health needs of young adults, through studying health literacy, is at the core of promoting health and preventing future diseases. The following subsections will address the interpretation of the current results within the context of the literature.

### The overall level of health literacy

Although there is no cutoff score for determining limited health literacy using the HLQ, these results revealed some of the limitations of health literacy among college students in Jordan. The lowest averages for the scales with possible total scores of (one to four) and (one to five) were regarding the scales “Feeling understood and supported by healthcare providers” and “Navigating the healthcare system”, respectively. A low score on the former scale indicates an inability to engage with and lack of having healthcare providers [7]. When the score of the latter scale is low, it reflects individual failure to advocate on his/her own behalf and the inability to find help using healthcare systems. Collectively, low scores on both dimensions negatively affect availability and access to valid health information pertinent to someone’s health needs.

### Mean differences based on demographic characteristics

The MANOVA results showed that age, gender, smoking status, year of study, and field of study all affect the linear composite of the dependent variables (HLQ scales) in college students. Based on the results of the univariate and follow-up analyses, age did not have any detected effect on the level of health literacy. There is evidence in the literature about the effect of age on health literacy among college students, measured using S-TOFHLA [18]. However, administering the HLQ to college students revealed similar results to those reported in this study, with a significant effect of age only on the “Appraisal of health information” scale [24]. Similarly, age had a weak, positive correlation with the same scale of the HLQ in a study conducted in Denmark [25]. The effect of another related factor, year of study, revealed differences in the levels of some HLQ scales. Of most importance, the results showed that first-, second-, and third-year students had lower levels of health literacy in the following scales: “Feeling understood and supported by healthcare providers”, “Appraisal of health information”, and “Understand health information”. This finding is consistent with the findings reported by other studies conducted using the HLQ [26] and the Adult Literacy Scale [27]. Further investigation is needed to explore how health literacy is affected by the year of study.

Regarding gender, the related literature provides mixed evidence regarding the relationship between college students’ gender and health literacy. The findings of this study showed that female students had higher levels of health literacy than males in the following areas: “Social support for health”, “Navigating the healthcare system”, “Ability to find good health information”, and “Understand health information” (Table 5). This is congruent with previous research findings for a study conducted among college students in Texas [24]. In contrast, a study conducted in China showed that male students had higher levels of health literacy than females [26]. Another study revealed no gender difference except for the “Social support for health” scale of the HLQ [25]. Such discrepancies could be attributed to different factors, including the variations in the educational systems and the sociocultural characteristics of college students [25]. Another possible explanation of the variation could be linked to the existing differences between the target populations. Whereas the target population of the current study was all college students (health-related and others), other studies targeted only health-related college students [25, 26]. Despite these mixed findings, gender should be considered a source of health literacy disparity in college students. This could be useful for creating customized programs for improving health literacy among college students.

Our results suggest that college students’ smoking status is a significant determinant of health literacy. Compared to smokers, nonsmokers had a higher level of health literacy on seven out of the nine scales of the HLQ. A recent study addressed the importance of studying campus tobacco culture in determining health literacy among college students [39]. According to the Healthy Campus 2020 [40], a campus-wide healthy environment is enhanced by adopting an ecological approach that focuses on both community and individual determining factors of health. Creating and/or activating smoking-free policies is paramount. Moreover, understanding the health needs of college students who smoke, as reported in this study, provides invaluable information to build a healthy campus.

Many research studies report the overall level of education as a predictor of health literacy [28, 30, 41]. In this study, we intended to investigate a usually overlooked area: the effect of students’ fields of study on health literacy. The field of study, health-related vs. others, significantly affected all scales of the HLQ. Those in the health-related faculties averaged significantly higher scores than students from other faculties on all scales. College classification was reported as a significant predictor of college students’ health literacy [18]. The number of health education topics studied by college students was found in previous research to be positively correlated with the level of health literacy [21]. Elsborg and colleagues found that public health students had higher scores on the HLQ scales than students from other health-related faculties [25]. These findings are, however, inconsistent with the findings reported by Zhang et al., who found that engineering students had higher scores on HLQ scales [26]. Both the levels of significance and the calculated effect sizes reported in this study indicate a disparity that should be considered in studying health literacy in college students. The potential implications of this particular area are discussed in the following section.

### Implications for health promotion and policy

The multidimensional nature of health literacy necessitates collaboration of efforts and utilization of the best available evidence about how it impacts health. However, evidence from the literature shows that health literacy is still an under-investigated phenomenon, especially outside the U.S. The larger study we are currently working on aims to improve the health promotion of college students and staff. Encouraging young populations, such as college students, to live a healthy lifestyle requires in-depth understanding of their health needs to build suitable health promotion interventions. In addition, a successful health promotion program is one that incorporates individual- and community-based information to create a healthy environment [40]. The results of this study highlight the disparities in health literacy as evidenced by variations based on the students’ age, gender, smoking status, year of study, and field of study. These results can be used to guide future investigations of the most effective intervention programs to enhance students’ health literacy and overall health.

A prominent finding of the current study is the lower level of health literacy in students from non-health-related faculties. Those students have apparent weaknesses in all dimensions of health literacy. The WHO’s publication *Framework for Action on Interprofessional Education & Collaborative Practice* calls for action to achieve better health through investing in interprofessional education [42]. Interprofessional education takes place when students from two or more fields learn about, with, and from each other [42]. Utilizing the resources available in educational institutions, health educators are encouraged to collaborate and create mandatory/elective courses and make them available to students from both health-related and non-health-related faculties. Such courses should be created considering the health needs of college students. Innovation in education through integrating evidence-based information with creative teaching strategies, such as project- and problem-based learning, seems promising. As the students work and learn together and from each other, the health outcomes will most likely be improved [42]. Additionally, the role of students from non-health-related faculties in disseminating educational and health outcomes is pivotal.

### Limitations

Despite the limitation of relying on self-report measures, combining the data from the demographics questionnaire with the HLQ provided a broader range of information regarding college students’ health literacy. We tried to strengthen our research by obtaining and integrating data regarding the anthropometric measurements from college students. Our intention was to measure such fundamental parameters as weight, height, and skinfold. However, that could not be accomplished during the current phase of enrollment in the larger study due to an inability to secure an appropriate, private space to take these measurements. Our intention is to gather such data and integrate it into future research about health literacy and promotion. Regarding the sample, we employed two essential criteria during the recruitment of participants, as discussed earlier, to enhance the representativeness of the study sample for the overall university population. However, some groups were not equivalent, for example, the year of study (fifth-sixth year) and smoking status, which could impact the generalizability of the findings. The small number of international students who participated in this study raises concerns about the generalizability of our findings to this group of college students. In addition, using a nonprobability sampling method, purposive sampling, could limit the generalizability of the findings.

## Conclusion

A successful health promotion approach is one that emphasizes the key role of disease prevention and health improvement starting with populations with fewer chronic health problems, such as school and college students. The results of this study showed that college students’ health literacy is influenced by demographic characteristics. Using the HLQ, we were able to identify the main health literacy needs and weaknesses of college students. The results could be used to guide future investigation regarding creating and examining the effectiveness of tailored interventional programs to improve college students’ health literacy. Finally, the disparity in health literacy based on the field of study provides promising information regarding the usefulness of interprofessional education in optimizing the health outcomes of college students.

## Supplementary information

**Additional file 1.** an English version of the demographics questionnaire.

**Additional file 2.** Distribution of the Health Literacy Questionnaire Scales.

## Data Availability

The datasets used and/or analyzed during the current study are available from the corresponding author on reasonable request.

## Abbreviations

ACHA: American College Health Association
ACHA-NCHA: American College Health Association-National College Health Assessment
AE: Ability to actively engage with healthcare providers
AMH: Actively managing my health
CA: Appraisal of health information
CDC: Centers for Disease Control and Prevention
CI: Confidence interval
DV: Dependent variable
EU: European Union
FHI: Ability to find good health information
HLQ: Health literacy questionnaire
HPS: Feeling understood and supported by healthcare providers
HSI: Having sufficient information to manage my health
IRB: Institutional Review Board
IV: Independent variable
MANOVA: Multivariate analysis of variance
NAAL: National Assessment of Adult Literacy
NHS: Navigating the healthcare system
QOL: Quality of life
SD: Standard deviation
SS: Social support for health
S-TOFHLA: Short Test of Functional Health Literacy in Adults
UHI: Understand health information
WHO: World Health Organization
