# Supplementary material for: Health literacy: exploring disparities among college students
Source: BMC Public Health. 2019 Oct 29;19:1401. doi: 10.1186/s12889-019-7781-2 (PMC6819582; doi:10.1186/s12889-019-7781-2)
Supplement: Supplementary file 1 — Additional file 1. an English version of the demographics questionnaire. [file 12889_2019_7781_MOESM1_ESM.docx]

**Demographics Questionnaire**

What was your age on your last birthday? _______________ years

ID #: _____________

What is your gender? Male

Female

What is your occupation?

Student

Faculty:………………………………………….

Specialty:………………………………………..

Employee

Unit:…………………………………………….

Experience:……………….. years

Faculty member

Faculty:………………………………………….

Department:………………………………………..

Experience:……………….. years

What is the highest level of education you reached?

Less than high school

High school

Bachelor's degree

Master's degree

Doctoral degree

Are you smoker? No

Yes

If you smoke, how long have you been smoking?

………………….. (years) and ………………. months

If you smoke, what type do you use?

Cigarettes, ………………… /day

Cigar, ……………………… /day

Electronic cigarettes, …………………. hour/day

Hookah/shishah, ……………………… hour/day

Other,………………………………….

Do you have any allergies? No

Yes

What causes your allergies?

Foods, including ………………………………….

medications, including ……………………………

Drinks, including…………………………………..

Other, ………………………………………………

Do you have any chronic diseases? No

Yes, name the disease(s)…………………… ………………...……………………….……

Do you take all the required vaccines? No

Yes

Do you take any prescribed medications? No

Yes, …………………………………… ………………………………………....

Do you take any over the counter medications? No

Yes, ……………………………… ………………………………………....

Are you interested in performing exercises on-campus?

No Yes

Do you think there must be places/facilities to perform light intensity exercises on-campus?

No Yes

If your answer to the previous question was “Yes”, where should such places/facilities be located?

Medical faculties, where exactly? …………………………………….

Engineering faculties, where exactly? …..……………………………

Classrooms building, where exactly? …………………………………..

The library, where exactly? ………………………………………

Other, where exactly? ………………………………………

Do you use the on-campus gym? No

Yes

If your answer to the previous question was “No”, what prevents you from using the on-campus gym?

Lack of time

Location of the gym

Lack of information about the gym

Financial reasons

Other, …………….………………………………………
