# Supplementary material for: Health literacy: exploring disparities among college students
Source: BMC Public Health. 2019 Oct 29;19:1401. doi: 10.1186/s12889-019-7781-2 (PMC6819582; doi:10.1186/s12889-019-7781-2)
Supplement: Supplementary file 2 — Additional file 2. Distribution of the Health Literacy Questionnaire Scales. [file 12889_2019_7781_MOESM2_ESM.docx]

| **Distribution of the Health Literacy Questionnaire Scales** |
| --- |
| 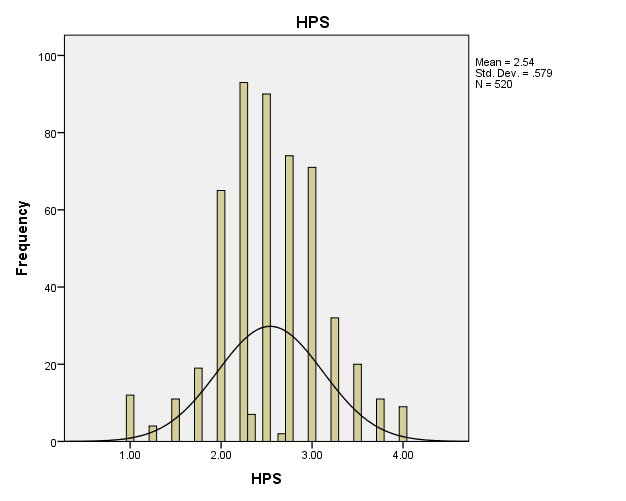 |
| 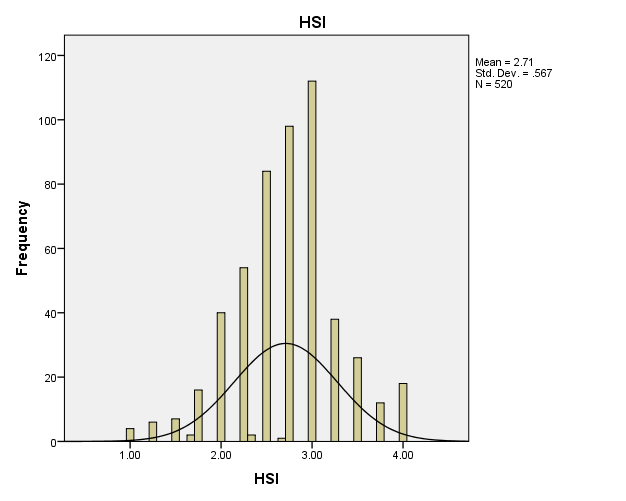 |
| 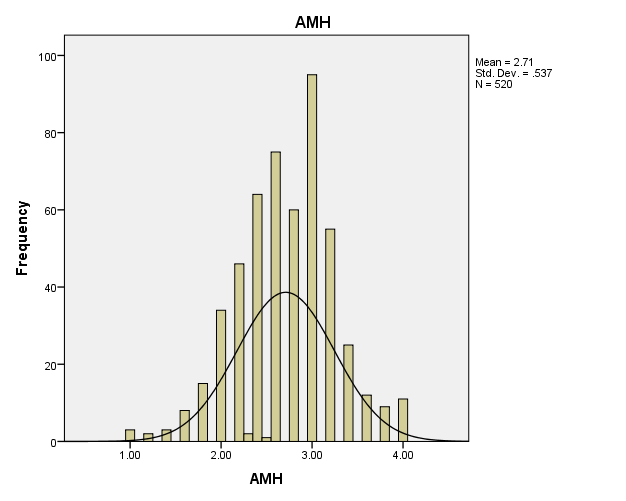 |
| 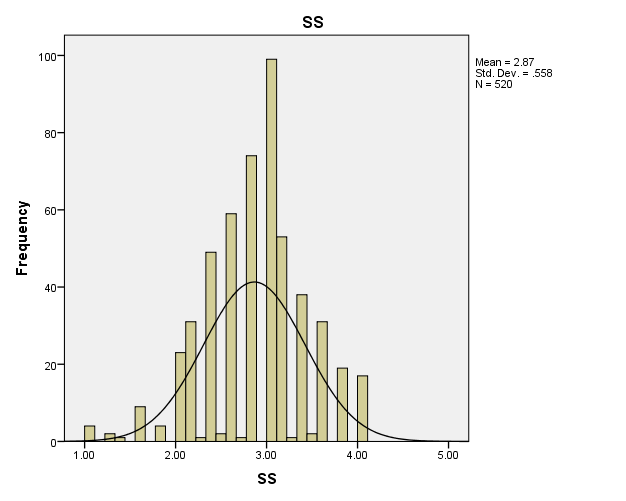 |
| 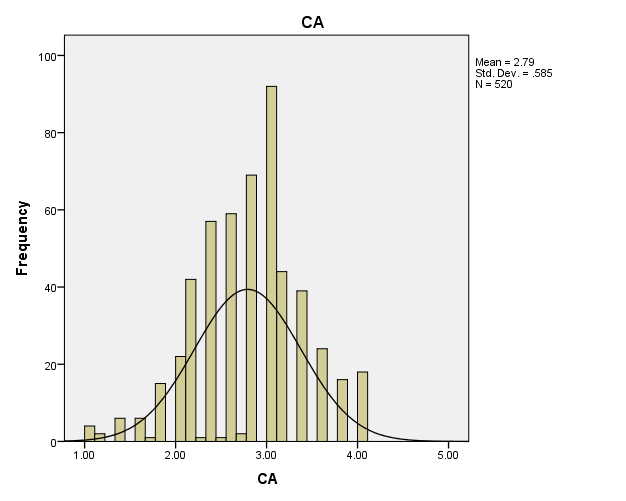 |
| 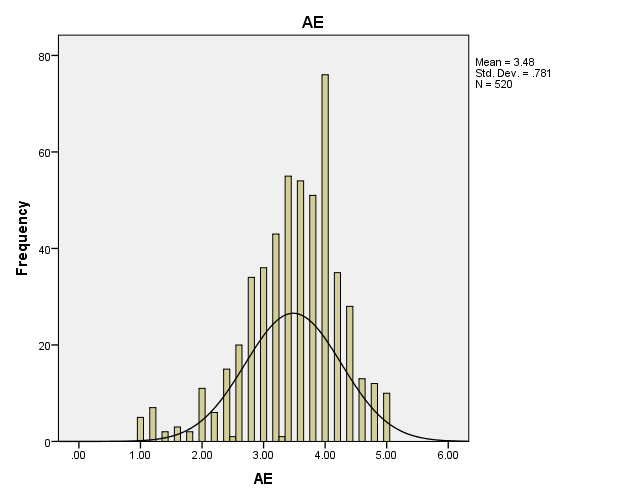 |
| 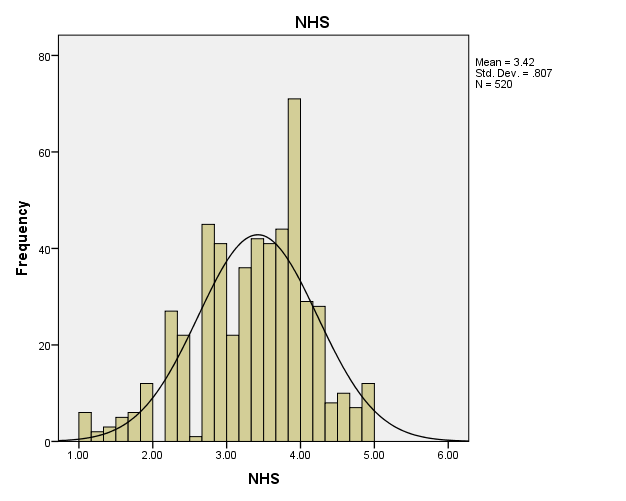 |
| 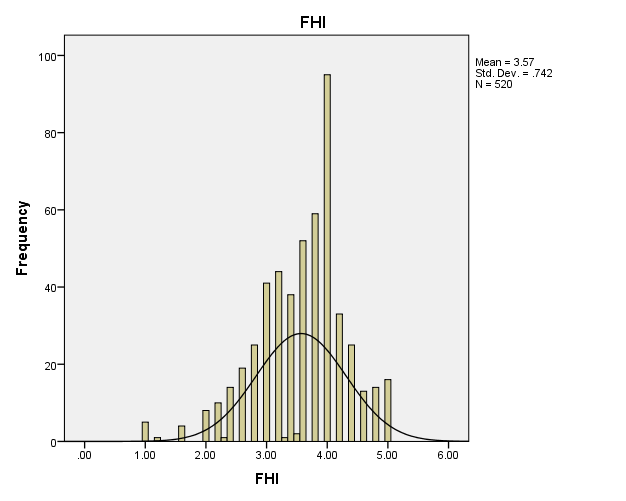 |
| 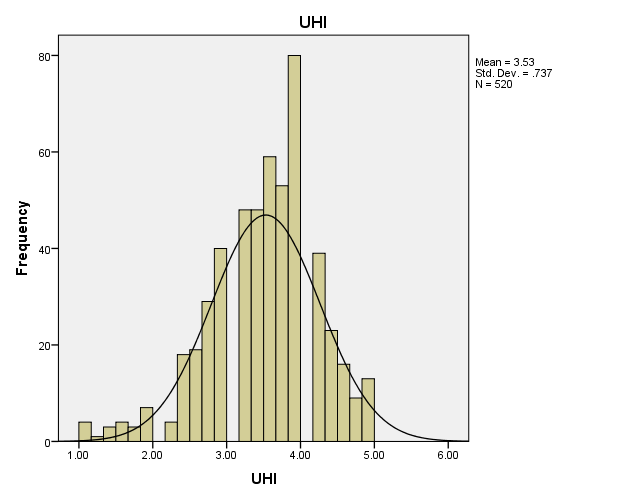 |
| Abbreviations: HPS = Feeling understood and supported by healthcare providers, HSI = Having sufficient information to manage my health, AMH = Actively managing my health, SS = Social support for health, CA = Appraisal of health information, AE = Ability to actively engage with healthcare providers, NHS = Navigating the healthcare system, FHI = Ability to find good health information, UHI = Understand health information |
